# Estimating the Additional Greenhouse Gas Emissions in Korea: Focused on Demolition of Asbestos Containing Materials in Building

**DOI:** 10.3390/ijerph13090902

**Published:** 2016-09-12

**Authors:** Young-Chan Kim, Won-Hwa Hong, Yuan-Long Zhang, Byeung-Hun Son, Youn-Kyu Seo, Jun-Ho Choi

**Affiliations:** 1 Department of Civil and Environmental Engineering, University of Michigan, 2350 Hayward St., G.G. Brown Building, Ann Arbor, MI 48109, USA; yyoungchani@gmail.com; 2 School of Architecture, Civil, Environmental and Energy Engineering, Kyungpook National University, 80 Daehak-ro, Buk-gu, Daegu 41566, Korea; hongwonhwa@gmail.com (W.-H.H.); showme0123@hotmail.com (Y.-L.Z.); s0913@knu.ac.kr (Y.-K.S.); 3 Department of Architecture, Daegu Technical University, 205 Songhyen-ro, Dalseo-gu, Daegu 42734, Korea; bonia96@naver.com; 4 Department of Fire Protection Engineering, Pukyong National University, 45 Yongso-ro, Nam-gu, Busan 48513, Korea

**Keywords:** greenhouse gas (GHG), asbestos containing materials (ACM), demolition stage, building

## Abstract

When asbestos containing materials (ACM) must be removed from the building before demolition, additional greenhouse gas (GHG) emissions are generated. However, precedent studies have not considered the removal of ACM from the building. The present study aimed to develop a model for estimating GHG emissions created by the ACM removal processes, specifically the removal of asbestos cement slates (ACS). The second objective was to use the new model to predict the total GHG emission produced by ACM removal in the entire country of Korea. First, an input-equipment inventory was established for each step of the ACS removal process. Second, an energy consumption database for each equipment type was established. Third, the total GHG emission contributed by each step of the process was calculated. The GHG emissions generated from the 1,142,688 ACS-containing buildings in Korea was estimated to total 23,778 tonCO_2_eq to 132,141 tonCO_2_eq. This study was meaningful in that the emissions generated by ACS removal have not been studied before. Furthermore, the study deals with additional problems that can be triggered by the presence of asbestos in building materials. The method provided in this study is expected to contribute greatly to the calculation of GHG emissions caused by ACM worldwide.

## 1. Introduction

### 1.1. Background

The Intergovernmental Panel on Climate Change (IPCC) has warned that without global efforts to reduce additional greenhouse gas (GHG) emissions, the mean global temperature may rise by up to 3–5 degrees by 2100 [1,2]. Thus, the global society is adopting extensive practices and policies toward reducing GHG emissions [3]. Various studies conducted over the past couple of years have focused specifically on the reduction of GHG emissions from the building sector [4,5,6,7], as this sector accounts for approximately 30% of total global GHG emission [8]. GHGs are generated by buildings directly and indirectly over the course of a building’s life cycle, from the construction stage through the operation demolition stages [9,10]. While many studies have focused on reduction of GHG emission during the construction and operation stages, there have been relatively few studies regarding GHG generated during the demolition stage [11,12]. However, demolition studies are rapidly becoming more relevant and more important. Building demolition in Korea is estimated to increase constantly in the future, as many of the country’s buildings were built in the 1960s and becoming aged [13]. To achieve Korea’s GHG reduction goal of 37% below business-as-usual (BAU) by 2030 [14], studies of GHG production during building demolition are of paramount importance. Asbestos has been in use since ancient times and, being prized for its physicochemical properties and affordability, saw a rapid increase in utilization after the industrial revolution [15,16]. Asbestos containing materials (ACM) fulfill approximately 3000 different purposes [17], 90% of which are as building materials [18]. However, a further study in the 1960s identified asbestos as a carcinogen that, following a latent period of between twenty to fifty years from the time of exposure, causes malignant mesothelioma, poor-prognosis lung cancer and pulmonary asbestosis [19,20]. In the 1990s, the use of asbestos was variously banned or limited by the global society due to human health effects caused by long-term exposure [21,22]. However, there are still many ACMs remaining in buildings today. In the United States, a reported 840,000 public and commercial buildings, including schools, contain asbestos [23]. In the 1970s, about 96% of imported asbestos was used in manufacturing asbestos cement slates (ACS), which were used as roofing material for building restoration projects led by the Korean government [24]. ACS still exists in approximately 17% of buildings in Korea today [25]. Weathering of remaining ACMs over time releases asbestos fibers into the environment and results in environmental air pollution and adverse health effects to the population [26]. For these reasons, the government of Korea is striving to fully remove ACS from all buildings.

### 1.2. Purpose and Method of the Research

**Figure 1:**
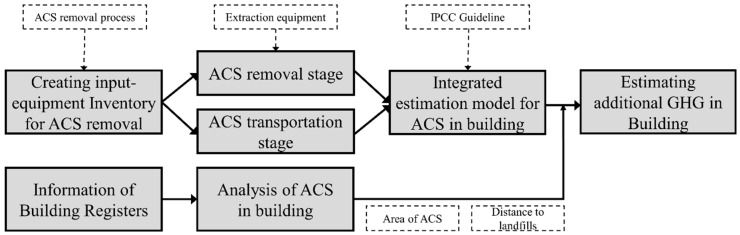
Overview of the methodology applied in this study to achieve Objective 1: developing an integrated estimation model; and Objective 2: calculating the total greenhouse gas (GHG) emissions generated by asbestos cement slates (ACS) removal for all of the buildings in Korea.

Building demolition can lead to emission of dust (e.g., hazardous fiber) into the environment [27]. Asbestos fibers can also be released into the environment during the removal process, causing the same deleterious environmental and health effects that the removal was commissioned to curb. Most at risk, in this case, is the health of demolition workers and the general public [27]. The EPA’s National Emission Standards for Hazardous Air Pollutants (NESHAP), the Occupational Safety and Health Act (2009), and the Asbestos Safety Management Act (2012) state that all ACM must be removed from buildings in advance of demolition [28,29]. Therefore, the building demolition process generates GHG emissions two-fold—first by the precursory step of ACM removal, and second, by the demolition itself. Because no existing studies have explored this additional GHG emission, the objectives of this study were to (1) develop a model for estimating the additional GHG generated by ACS removal during building demolition and (2) use this model to estimate the total GHG emissions generated by ACS removal in the entire country of Korea. An overview of the methodology is presented as a flow chart in Figure 1. This study is meaningful in that it discusses problems regarding GHG emissions that have thus far not been considered within the building sector. The methodology developed in this study allows the estimation of GHG emissions that were previously not accounted for in GHG modeling. The methodology can be used to produce databases of ACS removal emissions, which can, in turn, inform solutions and policies toward global GHG reduction in the future.

To achieve Objective 1, an integrated GHG estimation model was developed. First, regulations regarding ACS removal were considered and all removal processes were identified. An input-equipment inventory was created for each of the removal processes. Second, any equipment present in the inventory that is known to generate GHG emissions was assigned to one of two stages, removal stage or transportation stage, before defining its electricity and fuel consumption requirements and arranging the figures a database (DB). Third, an integrated estimation model was developed by applying the IPCC GHG calculation method to both the removal and transportation processes, and then summing them together. To achieve Objective 2, the total GHG emission generated by ACS removal was calculated for all of the buildings in Korea using the integrated estimation model developed in Objective 1. First, all buildings containing ACS were identified in each local governing unit using the building register. Second, the total area and weight of all ACS in each local governing unit was calculated, in addition to the total distance to landfills. The resulting values were compiled for all local governing units and applied as inputs into the integrated estimation model.

## 2. Theoretical Consideration and Application

The following section focuses on the theory of creating an input-equipment inventory based on an extensive literature review. The local governing units of Korea, building registers and landfill locations are also introduced. Last, the methods defined by the IPCC for attributing GHG emission levels to equipment based are discussed, based on electricity and fuel consumption.

### 2.1. Description of the ACS Removal Process

The amount of asbestos imported into Korea increased from 74,000 tons (1976) to 88,000 tons (1995), until the trend was reversed, with imports declining to only 6500 tons (2005) [30]. Since the complete ban on the import and use of asbestos in 2009, the government of Korea has been striving to fully remove all ACM from buildings in the country [31]. The ACM comprise ACS (roof), tex (ceiling), bamlite (wall), and gasket (facility). In addition, 96% of the total asbestos imported in the 1970s was used to manufacture the ACS used for roofs, in particular, which rank the highest of all ACM use [25]. For this reason, the ACS were set as the main ACM target in the present study. All ACS in Korea were manufactured to the Korean Standard (KS), which requires a combination of about 90% cement and 10% chrysotile [32]. Table 1 presents the industrial standard dimensions of ACS produced in Korea.

**Figure 2:**
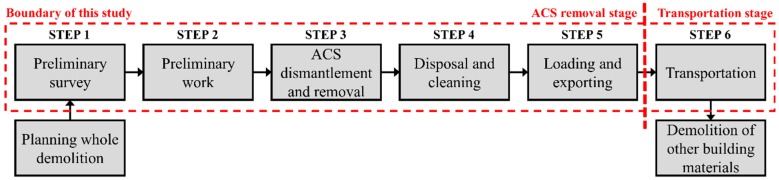
Demolition process of building and boundary of this study: (**1**) preliminary survey; (**2**) preliminary work; (**3**) ACS dismantlement and removal; (**4**) disposal and cleaning; (**5**) loading and exporting; and (**6**) transportation. If the result of Step 1 concludes that the building is ACM-free, the ACS removal process can be omitted, and the demolition can commence immediately.

It is stated by the Asbestos Safety Management Act (2012) that when demolishing a building (including remodeling), the inclusion of ACM must be surveyed in advance [29]. As a consequence, if the building contains ACM, the ACM must be removed before demolition [29]. Therefore, compared with the demolition of ACM-free buildings, additional GHG emissions occur during demolition of a building containing ACM. These extra emissions are generated by the electricity and fuel consumption of equipment used in the removal and transportation of the ACM prior to demolition. Therefore, the scope of this study was set as the entire ACS removal process of a building, from the preliminary survey to the transportation of ACS during the demolition stage. This study has classified the ACS removal process into six steps, based on asbestos-related laws, and results from the literature review [34]. Figure 2 depicts the scope of this study, inclusive of these six steps within the general demolition process of a building and the ACM removal process.

### 2.2. Assessing Method of GHG Emissions

GHG emissions for Korea are assessed in this study according to the IPCC method [35] and in accordance with the Korean Ministry of Environment’s guidelines for the operation of management by objectives concerning GHG and energy (Ministry of Environment Document No. 2013-180). The GHG emission levels generated by the electricity and fuel consumption of removal and transportation equipment were assessed using the IPCC classification system and the Ministry of Environment guideline document provided modifications suitable for the context of Korea. The guideline document suggested the emission factor of indirect electric power consumption, the equivalent coefficient of GHG, and both the net calorific value and the GHG emission factor according to fuel type. The guideline document provided a method for assessing GHG when using externally provided electric power (Tier 1), and a method for assessing GHG when using fuel (Tier 1). These methods are described in Equations (1) and (2):

(1) $$E_{j}=\underset{j}{\sum }\left(Q\times EF_{j}\times F_{eq,j}\right)$$

where *E_j_* is the GHG emission due to electric power consumption (tCO_2_eq); *Q* is the externally provided electric power consumption (MWh); *EF_j_* is the emission factor of indirect electric power consumption (tGHG/MWh); *F_eq,j_* is the CO_2_ equivalent coefficient of GHG (*j*) (CO_2_ = 1, CH_4_ = 21, N_2_O = 310); and j is the GHG type.

(2) $$E_{i,j}=\sum^{}\left(Q_{i}\times EC_{i}\times EF_{i,j}\times F_{eq,j}\times 10^{-9}\right)$$

where *E_i,j_* is the emission of GHG (*j*) by the type of fuel (*i*) (tCO_2_eq); *Q_i_* is the fuel consumption by the type of fuel (*i*) (ℓ); *EC_i_* is the net calorific value by the type of fuel (*i*) (MJ/ℓ); *EF_i,j_* is the emission factor of GHG (*j*) by the type of fuel (*i*) (kg/TJ); *F_eq,j_* is the CO_2_ equivalent coefficient of GHG (*j*) (CO_2_ = 1, CH_4_ = 21, N_2_O = 310); and *i* is the type of fuel.

### 2.3. Local Governing Units of Korea, Building Registers, and Landfill Locations

Building Registers [36] provide a summary of the information relating to all buildings in Korea [37]. The Building Register details are specified in the “Building Act” and the “Regulations on Registration and Management of Building Registers” in Korea. According to Article 38 of the Building Act, information related to the construction, maintenance and management of all approved buildings should be recorded and stored in the Building Register [31]. Information including the building location, name of building, use, lot number, building area, site area, total floor area, building volume-to-lot ratio, building coverage, structure, number of stories, height, roofing material, and date of approval for use, as well as the building owner’s name, ownership, and registration date are recorded and managed in the Building Register [25]. In this study, buildings with roof materials containing ACS were extracted from the building register, classified by local governing unit, and compiled in a database. However, there may be some discrepancy between this information and the actual status, due to the Building Register containing information only on legitimate buildings [38]. Nevertheless, from a practical aspect, this database has sufficient value because it is impossible to perform a field survey of all buildings.

**Figure 3:**
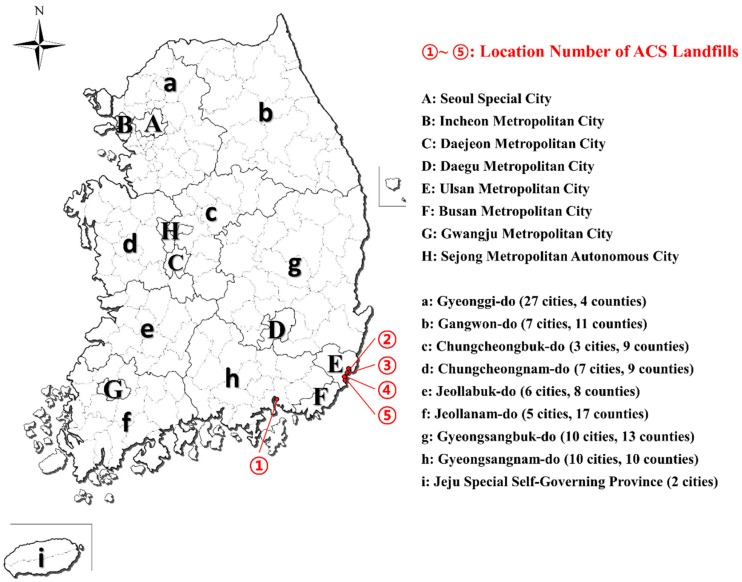
Local governing units of Korea. Metropolitan governing units (thick line, upper case), local governing units (thin line, lower case), and locations of ACS landfills are shown.

Administrative district of Korea is divided into metropolitan governing units and local governing units. The former are roughly classified into seventeen units (i.e., one special city, six metropolitan cities, one metropolitan autonomous city and nine provinces), and then further into 163 local governing units (cities and countries). The methods for the reclamation and disposal of ACM are currently under development [39]; however, the Wastes Control Act of Korea defines ACM as designated waste containing hazardous materials, which should be buried in places other than general landfills. As of 2013, a total of five locations existed specifically for burying ACM [40]. Additionally, each landfill contains a designated area for ACM burial. The majority of the ACM being buried are ACS. Figure 3 shows the local governing units in Korea, and the locations of ACS landfills.

## 3. Constructing the Database

This section describes the methods implemented in compiling the database (DB) that can later be utilized in calculating GHG emitted by ACS removal. First, an input-equipment inventory database was created, comprising all equipment utilized during the ACS removal processes (Step 1 through Step 6 of Figure 2). Next, the electricity and fuel energy consumption of this equipment, which causes GHG emission, were identified and added to the database. Finally, the Building Register was utilized to calculate the area and weight of ACS present in each local governing unit. These values were added to the DB along with the distance measures from the local governing unit to landfills.

### 3.1. Creating the Input-Equipment Inventory

In order to create the input-equipment inventory, initially, the six steps of ACS removal in Figure 2 were further divided into eighteen processes by observing all laws and guidelines related to ACS removal in Korea: for Step 1, asbestos survey and asbestos mapping; for Step 2, isolation; installation of hygiene equipment; vinyl installation on building exterior and floor; installation of vertical steel pipe scaffold; installation of horizontal steel pipe scaffold; and installation of safety net; for Step 3, personal protective gadget; spraying of chemical agents; measurement of asbestos density; and installation of packaging vinyl; for Step 4, ACS sealing and sticker placement and wet cleaning; for Step 5, post-measurement of asbestos density; sealing and sticker placement of other consumables; temporary storage and signboard installation; and for Step 6: ACS transportation. Next, the input-equipment inventory was established by identifying all equipment used in each of the 18 ACS removal processes [29]. Of this equipment, that requiring external electrical or fuel input was identified as shower equipment, drain filters, High Efficiency Particulate Air (HEPA) filter cleaners, and cargo trucks. Although asbestos samplers consume electric power, they were excluded from the list because they run on rechargeable batteries. The shower equipment and drain filter are hygiene equipment, which help the asbestos workers exit the worksite after work completion, while the HEPA filter cleaner is used for filtering the asbestos fibers from the worksite. The cargo truck is a transportation device for transporting ACS from the worksites to landfills. The complete input-equipment inventory, with electric-powered and fuel-driven equipment marked, is presented in Table 2.

### 3.2. Creating the Electric and Fuel Energy Consumption Database

The energy consumption of each electric-powered and fuel-driven equipment type (Table 2) was compiled into an energy consumption DB. The externally-provided electric power consumption (Q) requirement of electric-powered equipment type (shower equipment, drain filter, HEPA filter cleaner) was identified by survey. Each of the six enterprises that manufacture and sell such equipment were contacted via telephone surveys and visiting research to define the model number and electric power consumption of each equipment type. The results are shown in Table 3 [29]. In addition, the GHG emission factor of indirect electric power consumption (tGHG/MWh) based on the 2011 value published by the Korea Power Exchange (KPX) was used as shown in Table 4 [41].

Fuel consumption of fuel-driven equipment (cargo trucks) was expressed as distance traveled to landfill (derived in the following Section 3.3) divided by mileage. The average mileage for each tonnage of truck load was defined according to the 2011 values provided by the Korea Transport Institute, which is shown in Table 5 [42]. The fuel type (*i*) was set as diesel, and both the net calorific value of diesel (*EC_i_*) and the GHG emission factor (*EF_ij_*) were derived from the “Guideline for the operation of management by objectives concerning GHG and energy (Ministry of Environment Document No. 2013-180)”, which are reported in Table 6 [43].

### 3.3. Analysis of Building Registers and Distance to Landfills

Korean Building Registers were used to identify buildings containing ACS and establish their distance from landfills. First, all building registers in Korea were collected. The locations of buildings containing ACS roof materials were extracted from the registers and classified by local governing unit. It was found that the total number of buildings in Korea was 6,694,094, of which 1,142,688 (17.07%) contained ACS. Area information recorded in the building registers was utilized to calculate the area of ACS-containing buildings in units of m^2^/1000 for each local governing unit (Table 7).

The ACS removal process consists of two stages: the ACS removal stage and the transportation stage. The ACS removal stage is based on the area of ACS distribution, while the transportation stage is based on the load weight and transportation distance. However, building registers only report building area and roof materials. Thus, the presence of ACS and the building area were known, whereas the area of ACS application and total weight were not known. For this reason, it was necessary to develop a conversion for translating building area into ACS area. Once the area of ACS was deduced, the asbestos content and the weight of ACS per building could be calculated by utilizing Table 1 (asbestos content rate, weight per unit area). Generally, when calculating the roof area of a building, a conversion factor is used that considers the slope of the roof (1.3–1.6). In this study, a precedent study result of 1.428 was used in the calculation of the ACS roof area [44]. The method for calculating the area and weight of ACS per building area is illustrated by Equations (3) and (4). The total ACS area and weight contained in an entire local governing unit were estimated by applying Equations (3) and (4) to Table 7. Once the ACS weight was determined, the required number of cargo trucks for ACS transport could be calculated for each local governing unit:

(3) $$\text{Area}\;\text{of}\;\text{ACS}\;(\mathrm{kg})\;=\;1.428\times S_{A\;}$$

(4) $$\text{Weight}\;\text{of}\;\text{ACS}\;(\mathrm{kg})\;=\;(1.428\times S_{A})\;\times 10.5$$

where *S_A_* is building area.

To calculate the transportation distance, the minimum distance from the government of each local governing unit to an ACS landfill was determined using road network information within ArcGIS (Table 7). By utilizing the minimum distance and the average mileage from Table 5, the fuel consumption of each of the cargo trucks by the weight of the load was calculated.

## 4. Estimation of Additional GHG Emission

In this section, the model for estimating GHG emitted by ACS removal was developed using the electric and fuel energy consumption DB described in Section 3.2 and GHG emission factors. The model was developed in two stages: the ACS removal stage and the transportation stage. To develop the estimation model of the ACS removal stage, the GHG emission of electric-powered equipment was analyzed. To develop the estimation model of the transportation stage, the GHG emission of fuel-driven equipment was analyzed. An integrated estimation model was then defined by combining the two stages to predict GHG emissions and standardize by ACS area. Lastly, the integrated model was utilized to estimate the GHG emission of ACS removal in the entire country of Korea.

### 4.1. GHG Emission of Each Equipment Type

The GHG emission of the electric-powered equipment was calculated by applying the electric power consumption metrics (Table 3) and the KPX emission factor for 2011 to Equation (1), for each equipment type. To calculate the electric consumption of the shower equipment and drain filter, the average shower time per person was set as 21.4 min, while the running time of HEPA filter cleaner was set as 1 h per day [29,45]. The *Asbestos Safety Management Act* defines the area of ACS permitted to be removed in a day as 75 m^2^. Therefore, the GHG generated per equipment was divided by the area of ACS, to calculate the GHG emission per unit area (m^2^). Table 8 shows the results. The GHG emission of fuel-driven equipment was calculated via Equation (2). The cargo truck load weight, which corresponded to ACS weight, was converted into ACS area. GHG emission by distance (km) was calculated based on the average mileage for each ton of weight (Table 5). The GHG emission by distance (km) was divided by the area of ACS to calculate the GHG emission caused by the unit area (m^2^). The results are shown in Table 9.

### 4.2. Integrated Estimation Model Based on Unit Area of ACS

The final integrated estimation model predicting GHG emission per unit ACS area was developed by combining the calculations of GHG emissions during the ACS removal and transportation stages. To develop the estimation model for ACS removal, the GHG emission produced during the ACS removal stage (Table 8) was analyzed. The GHG emission produced by the combination of electric-powered equipment listed in Table 3, ranged from 1.0436 kgCO_2_eq to 2.7997 kgCO_2_eq. This was the total GHG emission for each equipment manufacturing enterprise and was considered as the average value of 1.8644 kgCO_2_eq for further use. The GHG emission based on unit area was then calculated by dividing this average value by ACS area. Thus, the estimation model of ACS removal is expressed as Equation (5):

(5) $${\mathrm{CO}}_{2}\mathrm{eq}\;\text{Emission}\;\left(\mathrm{kg}\right)=0.0249\;\left(\max:\;0.0373\;–\;\min:\;0.0124\right)\times {\;S}_{\mathrm{ACS}}$$

where CO_2_eq Emissions is the GHG emission of ACS removal stage; S_ACS_ is the area of ACS.

**Figure 4:**
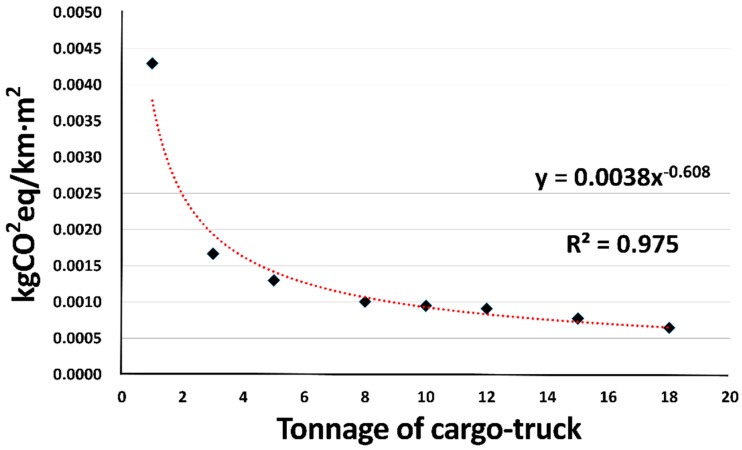
Diagram of GHG emission per weight of cargo truck in tons, based on units of ACS area.

To develop the estimation model for the transportation stage, the GHG emission per unit ACS area (kgCO_2_eq/km∙m^2^) for each ton of the cargo truck load (Table 9) was analyzed. A regression analysis was performed in SPSS (Statistical Package for Social Science) to analyze the relationship between GHG emission and weight of cargo load. The result was the GHG emission generated by the transport of 1 m^2^ of ACS over 1 km by a cargo truck. Table 10 and Figure 4 show the regression analysis results. As determined by the regression results, the GHG estimation model for the transportation stage can be expressed as Equation (6). An integration model was then derived by summing the estimation models for ACS removal and transportation:

(6) $${\mathrm{CO}}_{2}\mathrm{eq}\;\text{Emission}\;\left(\mathrm{kg}\right)=\left(0.0038\times T^{-0.608}\times M\right)\;\times {\;\mathrm{TS}}_{\mathrm{ACS}}$$

where CO_2_eq Emissions is the GHG emission of ACS transportation stage; T is the tonnage of cargo-truck; M is the distance to landfills; and TS_ACS_ is the area of ACS in each cargo-truck as Table 9.

## 5. Additional GHG Emission in Korea

**Figure 5:**
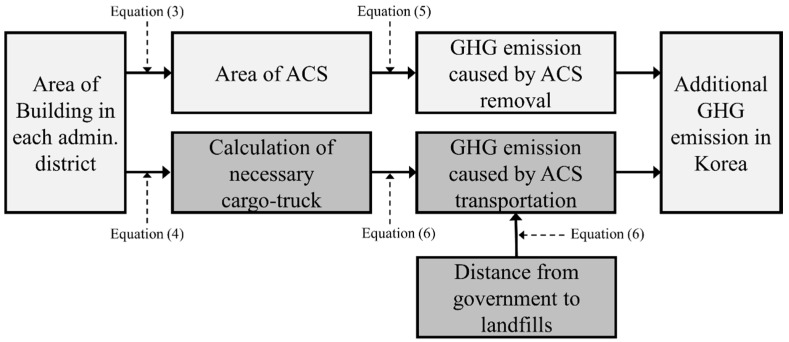
Flow chart representing the process of calculating GHG emission produced by ACS removal in the entire country of Korea.

The final step was to apply the integrated model developed in the previous sections to estimate the GHG emission that would be produced by ACS removal in the entirety of Korea. To accomplish this objective, buildings with roof materials containing ACS were extracted from building registers and classified by local governing unit. In addition, by utilizing building area information from the building register, the total area of all ACS-containing buildings was summed for each local governing unit (Table 7). By applying Equation (3) to the area of ACS-containing building, the area of ACS was calculated for each local governing unit. The total ACS weight per local governing unit was then calculated by applying Equation (4). The weight metric allowed determination of the number of cargo trucks required to transport the total load per local governing unit. By applying the ACS area within each local governing unit to Equation (5), the GHG emission generated during the ACS removal stage of each local governing unit was found. Next, the GHG emission generated during the ACS transportation stage was calculated by applying the required number of cargo trucks for each local governing unit and the distance to the landfills within Equation (6). Finally, the GHG emissions generated during the ACS removal stage and transportation stages were summed to estimate the total GHG emission caused by the full ACS removal procedure in the entire country of Korea. A representation of this methodological flow can be found in Figure 5.

The building registers indicated that the total number of buildings in Korea was 6,694,094, of which 1,142,688 (17.07%) contained ACS. The total ACS area included in the buildings of Korea was 169,144,378 m^2^. The entire weight of ACS in Korea was 1,776,016 tons. Depending on Figure 5, the total GHG emission produced by ACS removal and transportation for all of Korea was found to range from 2097 tonCO_2_eq to 6309 tonCO_2_eq (average 4212 tonCO_2_eq) and 21,681 tonCO_2_eq to 125,044 tonCO_2_eq, respectively. These results are summarized in Table 11. Summing both ACS removal and transportation figures, the predicted total GHG emission contributed by completed ACS removal procedures in the entire country of Korea ranged from 23,778 tonCO_2_eq to 132,141 tonCO_2_eq. Depending on the combination of equipment used to remove and transport ACS, the GHG emission generated in the ACS removal process could be reduced by up to 82%.

## 6. Conclusions

When demolishing a building, GHG emissions occur during the dismantling of general building materials. When ACM must be removed from the building before demolition, additional GHG emissions are generated. The GHG emission generated by the ACS removal stage per one day of ACS was found to range from 1.0436 kgCO_2_eq to 2.7997 kgCO_2_eq, while the GHG emission generated by transporting 1 m^2^ of ACS for 1 km by a single cargo truck ranged from 0.000646 kgCO_2_eq to 0.004298 kgCO_2_eq. The GHG emissions generated from the 1,142,688 ACS-containing buildings in Korea were estimated to range from 23,778 tonCO_2_eq to 132,141 tonCO_2_eq. A number of studies regarding asbestos have focused on considerations encompassing human toxicity, health risk, and optimal disposal. Meanwhile, previous studies about GHG have not considered the significance of the removal of ACM from buildings. Therefore, the significance of this study rested on the fact that previous studies have not investigated the emissions generated by ACM removal. Furthermore, the study dealt with additional problems triggered by the presence of asbestos in building materials. In further studies, because the transportation stage is actually a much greater contributor to GHG emissions than the ACM removal stage, efforts to find an optimal landfill site, and the option to combine ACMs with general construction waste, are likely to be important tools to reduce GHG emissions. Regardless of the problems that remain, the method provided in this study will contribute greatly to the ability to calculate the GHG emissions caused by ACM worldwide.

## Figures and Tables

**Table 1:** Type and characteristics of asbestos cement slates (ACS) used in Korea building: Slates used as roofing material were produced by two enterprises and included 100% asbestos.

Type of ACS		Width (mm)	Length (mm)	Content Rate (%)	Average Weight (kg/sheet)	Number of Corrugation	Depth of Corrugation (mm)	Weight/Unit Area (kg/m^2^)
Large Corrugation	No. 6	960	1800	Cement (90) and Chrisotile (10)	18	7.5	35	10.5
	No. 7	960	2100		21			
	No. 8	960	2400		24			
Small Corrugation	No. 6	720	1800		14	11.5	15	
	No. 7	720	2100		16			
	No. 8	720	2400		18			

These ACS were used for either small corrugation (residential use) or large corrugation (facility use). Chrisotile has been used more than other asbestos species (94% of the world’s production) [33].

**Table 2:** Input-equipment inventory for each ACS removal stage.

Rough Stage of ACS	Detailed Removal Process of ACS	Input-Equipment/Materials
Step 1: Preliminary survey	(1) Asbestos survey	Filter, Working clothes, Safety gloves
	(2) Asbestos mapping	
Step 2: Preliminary work	(1) Isolation	Warning sign, Safety belt, Tarpaulin, Shower *, Drain filter *, Sump, Vinyl sheet, Sanitation equipment, Floor vinyl, Steel pipe scaffold
	(2) Installation of hygiene equipment	
	(3) Vinyl installation on building exterior and floor	
	(4) Installation of vertical steel pipe scaffold	
	(5) Installation of horizontal steel pipe scaffold	
	(6) Installation of safety net	
Step 3: ACS dismantlement and removal	(1) Wearing personal protective gadget	Mask, Safety boots, Goggles, Special filter, Anti-dust garments, Anti-dust gloves, Sprayer, Working clothes, Asbestos sampler
	(2) Spraying of chemical agents	
	(3) Measurement of asbestos density	
	(4) Installation of packaging vinyl	
Step 4: Disposal and cleaning	(1) ACS sealing and sticker placement	HEPA filter cleaner *
	(2) Wet cleaning	
Step 5: Loading and exporting	(1) Post measurement of asbestos density	Filter, Working clothes, Safety gloves, Warning sign, Asbestos sampler
	(2) Sealing and sticker of other consumables	
	(3) Temporary storage and signboard installation	
Step 6: Transportation	(1) ACBM transportation	Cargo-Truck *

* Electric-powered and fuel-driven equipment are identified with asterisks. ACS removal steps 2, 4, and 6 generate the most greenhouse gas (GHG) emissions in the process. The remaining steps are conducted manually, resulting in no GHG emissions.

**Table 3:** Model number and electric power consumption of input-equipment.

Equipment/Company	Shower Equipment		Drain Filter		HEPA Filter Cleaner	
	Name of Equipment	Electrical Consumption (Watt)	Name of Equipment	Electrical Consumption (Watt)	Name of Equipment	Electrical Consumption (Watt)
A	LPS-80	7000	LDE-80	340	C-112S	1350
B	ASM	5000	AEWP3810	380	ASZ-201	2400
C	LT-110	6000	LT-C100	78	C-1125	1350
D	Ha-WS300	2500	HA-FS500	2500	KV-103S	2400
E	none	6000	none	1100	CK862H	2700
F	none	2500	none	600	Clean Zone	2000
Mean		4833.3		833		2033.3

Large capacity was set as the standard if the equipment was classified into large and small capacity [29].

**Table 4:** GHG emission factors for indirect electric power consumption in 2011 published by the Korea Power Exchange (KPX) [41].

Year	tCO_2_/MWH	kgCH_4_/MWH	kgN_2_O/MWH	tCO_2_e/MWH
2011 (Use)	0.4585	0.0052	0.0040	0.4598

**Table 5:** Average mileage of a cargo truck for each tonnage of load, derived from a study on plans for reducing economic freight transportation distance of mass transportation means, by Korea Transport Institute (2011) [42].

Tonnage of Truck	1 ton	3 ton	5 ton	8 ton	10 ton	12 ton	15 ton	18 ton
Fuel Efficiency (km/ℓ)	6.52	5.60	4.31	3.48	2.95	2.56	2.41	2.41

**Table 6:** Net calorific value of diesel (*EC_i_*) and GHG emission factors (*EF_ij_*) derived from the ”Guideline for the operation of management by objectives concerning GHG and energy (Ministry of Environment Document No. 2013-180)” [43].

Type of Fuel	CO_2_	CH_4_	N_2_O	Calorific Value
Diesel	74,100 (kg/TJ)	3.9 (kg/TJ)	3.9 (kg/TJ)	35.4 (MJ/ℓ)

**Table 7:** Analysis results of Building Resister and distance to landfills (1: Special City; 2–7: Metropolitan City; 8: Metropolitan Autonomous City; 9–39: Gyeonggi-do; 40–57: Gangwo-do; 58–69: Chungcheongbuk-do; 70–84: Chungcheongnam-do; 85–98: Jeollabuk-do; 99–120: Jeollanam-do; 121–143: Gyeongsangbuk-do; 144–161: Gyeongsangnam-do; 162–163: Jeju Special Self-Governing province).

No. 1–41				No. 42–82				No. 83–123				No. 124–163			
NC ^1^	AS ^2^	DL ^3^	NL ^4^	NC	AS	DL	NL	NC	AS	DL	NL	NC	AS	DL	NL
SeUl	1282	353	1	GaNe	215	299	3	TaAn	313	344	1	AnDo	1062	169	4
BuSa	2984	45	5	DoHa	86	259	3	DaJi	884	319	1	GuMi	1470	126	1
DaGu	2610	93	1	TaBa	172	256	3	JeoJ	186	194	1	YeJu	793	200	4
InCh	2943	364	1	SoCh	111	359	3	GuSa	904	235	1	Yche	1094	81	3
GwaJ	639	209	1	SamC	187	247	3	IkSa	786	215	1	SaJu	1579	165	1
DaJe	996	212	1	HoCh	275	331	1	JeEu	863	223	1	MuGy	833	182	1
UlSa	3796	9	3	HoeS	309	301	1	NaWo	852	147	1	GySa	1422	91	3
SeJo	628	241	1	YeWo	177	266	4	GiJe	1159	219	1	GuWi	528	132	3
SuWo	287	322	1	PyCh	224	298	4	WaJu	724	198	1	UiSe	1024	138	3
SeNa	122	329	1	JeSe	130	305	4	JiAn	328	159	1	ChSo	286	138	3
UiJB	242	362	1	ChWo	462	411	1	MuJu	200	163	1	YeYa	309	167	3
AnYa	458	339	1	HwCh	332	392	1	JaSu	437	150	1	YeDe	252	117	3
BuCh	248	362	1	YaGu	128	393	1	ImSi	497	169	1	ChDo	731	71	1
GwMy	269	353	1	InJe	233	383	1	Scha	651	175	1	GoRy	649	86	1
PyTa	1056	289	1	GoSe	96	383	3	GoCh	395	230	1	SeJu	652	99	1
DoDC	349	382	1	YaYa	112	344	3	BuAn	368	236	1	ChGo	513	110	1
AnSa	383	341	1	CheJ	317	231	1	MoPo	240	254	1	YeCh	967	190	4
GoYa	370	370	1	ChuJ	808	241	1	YeSu	1400	132	1	BoHw	718	210	3
GwCh	8	343	1	JeCh	603	262	4	SChu	915	132	1	UlJi	429	187	3
GuRi	120	346	1	CheW	782	231	1	Naju	1539	217	1	UlLe	7	78	3
NaYJ	1045	347	1	BoEu	640	200	1	GwYa	445	113	1	ChaW	1215	11	1
Osan	142	308	1	OkCh	738	194	1	DamY	741	191	1	JiJu	1334	62	1
SiHe	297	349	1	YeDo	702	170	1	GokS	436	166	1	ToYe	472	69	1
GuPo	51	337	1	JiCh	563	258	1	GuRy	301	144	1	SaCh	811	78	1
UiWa	81	334	1	GoSa	593	229	1	GoHe	941	183	1	GiHa	1485	32	1
HaNa	282	334	1	EmSe	797	247	1	BoSe	675	180	1	MiYa	1143	49	1
YoIn	1388	311	1	JePy	143	239	1	HwSu	605	190	1	GeJe	415	73	1
PaJu	1285	383	1	DanY	320	242	4	JaHe	594	201	1	YaSa	771	40	4
Iche	991	294	1	ChAn	1102	275	1	GaJi	720	217	1	UiRy	679	44	1
AnSe	778	284	1	GoJu	565	243	1	HaeN	1130	236	1	HaAn	895	27	1
GiPo	956	375	1	BoRu	203	281	1	YeAm	813	226	1	ChNy	764	48	1
HwSe	1531	331	1	ASan	463	280	1	MuAn	1280	240	1	GoSo	968	50	1
GwJu	1031	319	1	SeSa	456	326	1	HaPy	532	237	1	NaHa	827	108	1
YaJu	1371	368	1	NoSa	501	230	1	YeGw	380	247	1	HaDo	915	108	1
PoCh	2157	381	1	GyRy	20	225	1	JaSe	713	216	1	SanC	516	90	1
YeoJ	1591	289	1	GeSa	605	188	1	WaDo	839	268	1	HaYa	496	111	1
YeoC	661	405	1	BuYe	895	250	1	JiDo	597	281	1	GeCh	527	109	1
GaPy	331	368	1	SeCh	362	249	1	ShAn	971	260	1	HaCh	1043	72	1
YaPy	931	313	1	ChYa	338	278	1	PoHa	1443	73	3	JeJu	2397	19	1
ChCh	510	360	1	HoSe	516	301	1	GyJu	1355	47	3	SeGP	931	60	1
WoJu	399	284	1	YeSa	518	285	1	GiCh	975	132	1				

^1^ Abbreviation name of cities and countries (e.g., Seoul: SeUl, Busan: BuSa); ^2^ Area of ACS containing building (unit: m^2^/1000); ^3^ Distance to landfills (unit: km); ^4^ Location Number of landfills.

**Table 8:** GHG emission of electric-powered equipment (unit: kgCO_2_eq).

Equipment	Shower Equipment	Drain Filter	HEPA Filter Cleaner	Total GHG Emission	ACS Work Area per One Day	GHG Emission per Unit Area of ACS (/m^2^)
A	1.1481	0.0558	0.6208	1.8247	75 m^2^	0.0243
B	0.8201	0.0623	1.1036	1.9860		0.0265
C	0.9841	0.0128	0.6208	1.6177		0.0216
D	0.4100	0.4100	1.1036	1.9237		0.0256
E	0.9841	0.1804	1.2416	2.4061		0.0321
F	0.4100	0.0984	0.9197	1.4281		0.0190
Mean	0.7927	0.1366	0.9350	1.8644	75 m^2^	0.0249

**Table 9:** GHG emission of fuel-driven equipment (unit: kgCO_2_eq).

Ton of Truck	Fuel Efficiency (km/ℓ)	GHG Emission (/km)	Area of ACS (m^2^)	GHG Emission per Unit Area of ACS (/km∙m^2^)
1	6.52	0.4093	95.24	0.004298
3	5.60	0.4766	285.71	0.001668
5	4.31	0.6192	476.19	0.001300
8	3.48	0.7669	761.90	0.001007
10	2.95	0.9047	952.38	0.000950
12	2.56	1.0425	1142.86	0.000912
15	2.41	1.1074	1428.57	0.000775
18	2.41	1.1074	1714.29	0.000646

**Table 10:** Results of regression analysis between GHG emissions and weight of cargo truck load in tons, based on units of ACS area.

Model Summary					Parameter Estimates	
R Square	F	df1	df2	Sig.	Constant	b1
0.975	231.460	1	6	0.000	0.0038	−0.608

Sig.: significance probability.

**Table 11:** Estimated GHG emission caused by the removal of all ACS in Korea (unit: tonCO_2_eq).

**Ton of Truck**	1 ton	3 ton	5 ton	8 ton	10 ton	12 ton	15 ton	18 ton
**GHG emission of ACS removal**	4212 (max: 6309; min: 2097)							
**GHG emission of transportation**	125,832	64,399	47,210	35,482	30,982	27,735	24,220	21,681
**Total GHG emission (mean)**	130,044	68,611	51,422	39,694	35,194	31,194	28,432	25,893
